# Bio and health informatics meets cloud : BioVLab as an example

**DOI:** 10.1186/2047-2501-1-6

**Published:** 2013-02-04

**Authors:** Heejoon Chae, Inuk Jung, Hyungro Lee, Suresh Marru, Seong-Whan Lee, Sun Kim

**Affiliations:** 1 School of Informatics and Computing, Indiana University, Bloomington, Indiana USA; 2 Interdisciplinary Program in Bioinformatics, Seoul National University, Seoul, Korea; 3 Pervasive Technology Institute, Bloomington, Indiana USA; 4 Department of Brain and Cognitive Engineering, Korea University, Seoul, Korea; 5 Computer Science Department, Seoul National University, Seoul, Korea; 6 Bioinformatics Institute, Seoul National University, Seoul, Korea

**Keywords:** Bioinformatics, Cloud computing, Big data, Workflow, User interface, Data integration, Analysis, Security

## Abstract

The exponential increase of genomic data brought by the advent of the next or the third generation sequencing (NGS) technologies and the dramatic drop in sequencing cost have driven biological and medical sciences to data-driven sciences. This revolutionary paradigm shift comes with challenges in terms of data transfer, storage, computation, and analysis of big bio/medical data. Cloud computing is a service model sharing a pool of configurable resources, which is a suitable workbench to address these challenges. From the medical or biological perspective, providing computing power and storage is the most attractive feature of cloud computing in handling the ever increasing biological data. As data increases in size, many research organizations start to experience the lack of computing power, which becomes a major hurdle in achieving research goals. In this paper, we review the features of publically available bio and health cloud systems in terms of graphical user interface, external data integration, security and extensibility of features. We then discuss about issues and limitations of current cloud systems and conclude with suggestion of a biological cloud environment concept, which can be defined as a total workbench environment assembling computational tools and databases for analyzing bio/medical big data in particular application domains.

## Review

High throughput, massive parallel sequencing technologies, called the next generation sequencing (NGS) technologies, were first introduced in the late 1990s. The leap of sequencing technology by NGS dropped sequencing cost dramatically and caused the exponentially increasing volume of data. Comparing NGS to the traditional Sanger sequencing technology that was used for the first human genome project, a three billion dollar project over a span of 10 years, the cost and time for sequencing a single human genome has dropped by 3,000,000 and 3000 times respectively [1, 2]. As a result, the nucleotide sequence resource in the EMBL-Bank DB has approximately doubled over the past 5 years [3, 4]. The sequencing throughput rate has increased five fold per year, whereas computer performance generally follows the Moore’s Law, doubling only every 18 or 24 months. Thus the gap between the production rate of sequencing data and computing power to process them is widening. On January 10, 2012, the Life Technologies company announced the Benchtop Ion Proton Sequencer [1] claiming the opening of the US $1000 human genome sequencing era. The new sequencing technology is not only affordable in price, but also allows human genome to be sequenced within a single day compared to weeks or months with conventional technologies. In addition, the Pacific Biosciences company recently announced new technology which can sequence DNA fragments of several thousand bps in length [2]. The higher multi fold coverage with longer sequences will make human genome sequencing much easier and the quality of the sequencing data is also expected to be more accurate. Thus, it is increasingly likely that biological sequence information is used for clinical applications with more confidence.

### Importance of big and high quality data in bio/health informatics

The low cost, high quality sequencing data has begun to impact bio/medical research fields. Higher resolution of genomic information, acquired through the improved fold coverage, makes it possible to associate genomic features, such as single nucleotide polymorphism, gene copy number variations, genome re-arrangement, and gene fusion, with disease phenotypes. In addition, sequencing data makes it possible to measure epigenetic features within cells, such as DNA methylation, histone modification, and microRNA. By measuring comprehensive epigenetic events in cells, it is now possible to understand how important genes, such as cancer suppressors and oncogenes, are activated or suppressed in cells. Thus we have an opportunity to not only measure disease susceptibility but also make prognostic measurements for treatment outcomes. However, each genetic and epigenetic event is measured independently, thus, to realize these possibilities, it is critical to integrate data of multiple types from genetic and epigenetic events and information systems along with powerful bioinformatics tools that should serve the research and clinical communities.

### Challenges from biomedical big data and expensive computation

The two major challenges in terms of biological data analysis are how to obtain computation power and how to perform data analysis for the bio/medical big data. The increase in cost for analyzing large biomedical data has become a major bottleneck for initiating biological research projects, especially with the introduction of NGS technology that produces data multiple folds in size compared to conventional sequencing technologies. Since acquisition (or sequencing) of data is becoming easier, the trend is that biomedical researchers formulate complicated questions investigating relationship among genetic and epigenetic elements in multiple cells of different phenotypes. Comparing omics data from multiple cell lines will be a significant challenge since the computing power and information systems are barely handling omics data from a few cell lines. Bio/medical researchers are proactive in leveraging the sequencing data. According to a recent poll on biomedical research facilities, a significant number of research institutions are already performing multi-omics research projects and most of them are experiencing the capacity limit of their computing facilities. The current situation is “data acquisition is affordable but data analysis is not.”

The need for computing capacity in the bio/medical applications varies dramatically for different stages. For example, processing a huge omics data or assembling short reads to reconstruct genome sequences requires a very big computing power with huge storage space. However, steps following these computationally expensive applications may not require as much computing power in the previous steps. It implies that the computing resources do not need to be maintained at the maximum capacity at all times, which makes use of public computing facilities highly desirable. Although there has been significant effort to provide high performance computing service to the public domain, e.g., the Grid projects, most of bio/medical researchers are not comfortable with such service due to technical issues and due to the lack of configurability of the computing systems. In addition to the computing power and storage space, there are significant number of computational tools and several thousands of biological databases. Thus collecting and configuring suitable tools and resources for certain research purposes is non-trivial job, even for bioinformatics experts.

### Cloud computing as an emerging solution

As an affordable and efficient way to overcome the hurdles mentioned in the previous section, *cloud computing* is gaining attention. This is because a cloud delivers computing resources as service rather than a physical product. Softwares and shared resources are pre-installed and provided on the cloud for all users. Cloud is analogous to the Internet as it can be simply a space where information and resource is shared among users.

Cloud computing has been successful in delivering computing resource (e.g computation power, servers, storage, applications) in terms of three forms of services such as Software as a Service (SaaS), Platform as a Service (PaaS) and Infrastructure as a Service (IaaS). Among cloud computing service models, the Virtual Machine (VM) type provided as IaaS is most suitable for biomedical data analyses since researchers are mostly looking for computation power to analyze their big data. With the IaaS model, users can easily configure computer systems with the computing power necessary to analyze the data. Thus users can enjoy computing power and storage on a “pay as you go” basis. However, IaaS model provides only computing machines, thus users need to configure softwares and databases that are necessary for data analysis. This is a significant barrier for bio/medical researchers. Thus some cloud service providers try to provide pre-installed packages including necessary tools and databases. For example, Amazon provides many genomic resources such as NCBI sequence data and the 1000 genome project data pre-installed and also provide APIs to the NCBI data sources. While this does not provide a total solution, users’ burden to configure softwares and databases is certainly relieved. Alternatively, PaaS and SaaS can be more user friendly models. However, the field of bioinformatics changes very fast and PaaS and SaaS models have not been successful for bio/medical application since service providers need to constantly re-configure PaaS and SaaS based cloud systems. We will discuss the cloud service model issue later in depth. In addition to the service model issues, due to the nature of cloud computing being a third party service, cloud service is still being questioned for other issues such as security and system outage.

### Advent of cloud systems for bio/medical informatics

The availability of high resolution, high throughput data from the NGS technologies has begun to transform conventional biological sciences to data-driven sciences. A number of very large scale science projects were launched to measure and accumulate data for genetic and epigenetic events, rather than starting from specific scientific questions. Examples of such projects include Encyclopedia of DNA Elements (ENCODE) [5], modENCODE [6], The Cancer Genome Atlas (TCGA) [7], and the 1000 Genome project [8]. From the deluge of the NGS data, analyzing and mining this big data is the key issue of the current research trend. Since only a handful of large research organizations can afford computer facility to utilize such data, many researchers have turned their attention to the potential benefits of cloud computing to handle biological data.

Many bio/medical cloud systems have emerged and some systems began to meet increased biological and computational needs. Most bio/medical cloud systems provide simple command-line execution of scripts or pipelines to the user. Although they are systems for elementary, a single task, they are well-suited for the IaaS concept of cloud computing as users can configure systems with the capacity to analyze the data. Some bio/medical cloud systems are more tightly coupled with cloud resources. Existing non-cloud applications have been redesigned to launch service on the cloud by utilizing features of cloud computing such as flexible resource allocation on demands, automatic system configuration, cost efficiency, and unlimited resources.

Recently bio/medical cloud services have begun evolving from simple and primitive applications to offering integrated and complex systems. This is because researchers have begun to formulate comprehensive scientific questions by utilizing data from various cellular events. This requires integration of various tools and biological databases. To keep pace with this trend, the cloud service providers have widely used computational tools and databases pre-installed and also provided libraries that can be used to access data from the source directly. Thus users have begun utilizing bioinformatics cloud service not only without worrying about the manual system installation and data integration processes, but also with reduced data uploading/transferring time.

#### Survey of current bio/medical cloud systems

In this section we survey current bio/medical cloud systems in terms of user interface (UI), extensibility and data integration.

Taverna [9] is a workflow management system that provides interoperability between cloud and various types of research projects in multiple disciplines, such as astronomy, bioinformatics, chemistry and music composition. Through the Taverna web portal, users can upload their input data to the cloud and invoke multiple computing units. While the analysis is being performed, the user can monitor the progress and when the analysis is complete, the web portal gets notifications. Taverna also allows the user to define how the data flows between services or pipelines by composing a graphical workflow diagram. This is done by drag and dropping resources from specific function blocks. Optionally, Taverna allows workflows to be composed and run by using a command interface. Once a workflow is composed, it can be shared with other researchers for collaboration. Since Taverna’s usage is designed for multiple science disciplines, it is not tailored for biology applications and some biological tools may not be present, in which the user need to provide them. Nevertheless, in the bioinformatics community, many projects preferably used Taverna as the workflow supporting analysis tool.

FX [10] is another recently released cloud system that provides a user-friendly web-based interface to provide high usability of biological tools for users who are not familiar with softwares. Utilizing the cloud computing infrastructure, FX provides analysis tasks such as estimating gene expression level and genomic variant calling from the RNA-seq reads using transcriptome-based references. Based on the Amazon Web Service (AWS), it provides a web-based working environment where the user can upload data and configure analysis settings based on options that the system provides. The analysis steps are however not as flexibly arrangeable as the workflow composing systems, but may be set with specific parameters for each analysis task (e.g., hit count for SNP, INDEL or alignment options). Since it is designed for specific tasks, it doesn’t require manual arrangement of function pipelines. Parameter settings and execution of the analysis are done by using a web-interface.

Systems such as Galaxy [11] are designed to provide complex multi-functioned open research frameworks. Galaxy offers generalized tools and libraries as analysis components. With a functional built-in workflow composer followed by fundamental editing tools, Galaxy allows users to construct user-defined pipelines. Galaxy also has its own visualization functionality that shows the progression of the workflow. Instead of fixed analysis tools and purpose, users may contribute additional tools (i.e., pipelines or function blocks) to the Tool Shed repository provided by Galaxy, extending the functionality of Galaxy. The Galaxy Tool Shed enables creating and sharing of Galaxy tools across the Galaxy community. Galaxy integrates multiple biological DBs, such as UCSC table data, BioMart Central, and modENCODE server.

Similarly SeqWare [12] is also a large biological workbench that provide an open workframe like Galaxy. In terms of extensibility, SeqWare supports the tool extension function, allowing users to write pipeline modules in Java. SeqWare provides four tools specifically designed to be compliant with massive parallel sequencing technologies (e.g., Illumina, ABI SOLiD, and 454), a web application (i.e., Laboratory Information Management System (LIMS)), pipelines for processing and annotating sequenced data, a query tool (SeqWare Query Engine), and a MetaDB that provides a common database to store the metadata used by all components. The SeqWare MetaDB imports data from other DBs and adds the metadata for further analysis. Hence, different DBs can be integrated and semi-unified to enable utilization of such data using different tools (or pipelines) provided by SeqWare, without worrying about data handling at each analysis step.

DNAnexus [13] like Galaxy supports data integration features in the system. DNAnexus is designed for the next generation sequencing data management, and it is also integrated with LIMS systems that can directly pass the experiment metadata to the system. One of the key benefits of integrating the external data with the system is that it supports automatic data conversion, which is then easily processed by various analysis tools. Research utilizing data from multiple sources often suffers from incompatible data formats and types. Manual data conversion for integrated analysis is not only a huge burden and a time consuming task but it also prevents automation of analysis tasks. Hence, data integration, along with data conversion, is an important feature of bio/medical cloud systems.

Another cloud based system, BioVLab [14, 15], is coupled with a graphical workflow composer called XBaya [16]. We review the BioVLab architecture later to provide detailed discussion on the design features and also to discuss the concept of the environment.

There are bio/medical cloud systems that serve as a virtual operating system with pre-installed packages and libraries required for analysis. CloVR [17] is a virtual machine with a specific purpose for analyzing biological data, such as large-scale on-demand BLAST searches, whole-genome assembly, gene finding and 16S ribosomal RNA sequence analysis. CloVR is implemented as a standalone workbench and an online application over the Internet. However, CloVR does not provide a good UI like workflow composers. Instead, CloVR provides command-line based automated analysis pipelines using pre-configured software packages for composing workflows. Such pre-configured software packages may be arranged in sequential or parallel manner as a workflow for analysis and it can be executed within a single virtual system. CloudBioLinux [18] is a Linux distribution with a pre-installed analysis packages and libraries available as an image loaded on the cloud. As an open source project, researchers and developers can extend the pool of libraries and packages.

We now survey cloud based systems that are designed for single or a few tasks rather than for providing analysis environments. Examples of systems of this type are CloudRSD, the cloud version of Roundup [19], CloudBurst [20], and Crossbow [21]. CloudRSD is a reciprocal smallest distance algorithm to compute orthologs using RSD on the cloud. It uses the maximum likelihood of the evolutionary distance to compute an ortholog score.

Cloudburst is a cloud version of RMAP, a well-known short-read alignment tool. It utilizes the MapReduce [22] framework, Hadoop [23], to map short reads to reference genomes in a parallel fashion utilizing the cloud environment. Cloudburst allows any number of mismatches or differences, and reports either all alignments or the best alignment for each read.

Crossbow is a package consisting of Bowtie [24], a fast and memory efficient read aligner, and SoapSNP [25], a consensus sequence builder and genotyper. It uses Amazon’s Elastic MapReduce [26] for parallel computation. It is shown that Crossbow can analyze over 35 fold the coverage of human genome in three hours with a 320-core cluster on the Amazon cloud.

eCEO designed and implemented a Cloud-based Epistasis cOmputing (eCEO) model for large scale epistatic interaction in genome-wide association study (GWAS) [27]. GWAS study needs to consider a large number of combinations of SNPs, which requires huge computing power. The eCEO model has shown its power to compute GWAS in terms of phenotypes using cloud computing.

Table 1 shows comparison of features of cloud-based bio/medical applications. Features that are considered for comparison among each application are: ‘Graphical UI’ for the frontend graphical interface, ‘External data integration’ for the availability of external data or databases for analysis, ‘Security’ for security features used for securing data or transactions of analysis processes, ‘Extensibility’ for the system feature that allows users to implement their own analysis functions in extend to the cloud application and ‘Cloud usage’ for adopting the cloud system.

**Table 1:** **Comparison of bio/medical cloud systems**

Cloud application	Graphical UI	External data integration	Security	Extensibility	Cloud Usage
BioVLab	Xbaya	O	MyProxy, AWS Credential	GFac	Pre-built system
Cloudburst	-	-	AWS Credential	-	Parallel processing
Cloud Bio Linux	-	-	AWS Credential	-	Pre-built system
Cloud RSD	-	-	AWS Credential	-	Parallel processing
CloVR	CloVR portal	-	AWS Credential	-	Pre-built system
Crossbow	-	-	AWS Credential	-	Parallel processing
DNAnexus	DNAnexus Web	O	ISO 27002	-	Computing resource
Galaxy	Galaxy Web	O	AWS Credential	Tool Shed	Pre-built system
Myrna	-	-	AWS Credential	-	Parallel processing
SeqWare	SeqWare Portal	O	GnuPG	Java Modules	Computing resource
Taverna	Taverna Workbench	O	WS-Security	API consumer tool	Interoperability

This is a comparison table of bio/medical cloud systems in terms on their features.

### Bio/medical cloud environment

In this section, we discuss design issues of bio/medical cloud systems and then introduce a concept of “cloud environment” for bio/medical applications.

#### Limitation of current bio/medical cloud systems

Although cloud-based bioinformatics systems are gaining attention, there are several important system design issues that should be resolved to be useful in practice. System usability is the main system design issue for bio/medical cloud systems. Although many cloud systems relieve users’ burden for system configurations and try to improve system usability, it is still challenging to perform complicated analysis tasks by combining multiple tools and databases. Handling big input data is an open problem. Delivering analysis results (output from the analysis tasks) is also an open problem that needs to be studied extensively.

Another important issue comes from a plethora of bioinformatics resources and the breadth of the bio/medical fields. Since there are too many bioinformatics tools and databases, it is very difficult even for bioinformatics experts to identify which tools can be used for specific research or clinical purposes, locate, install, and assemble them. As of now, there is little effort to handle this situation. System extensibility is also an important issue. For example, consider a situation where a scientist believes that a new bioinformatics tool is most suitable for a scientific goal. How can she/he hook in the tool to existing bioinformatics cloud systems?

Some bioinformatics cloud systems are designed to address these issues and we will describe BioVLab systems that use a graphical workflow composer, as an example of systems whose design goals are to address these issues.

#### BioVLab: Virtual Collaborative Lab for Bio/medical Applications

BioVLab [28] is a computational infrastructure on the cloud, coupled with a graphical workflow composer. It is a three layer architecture where a graphical workflow composer and web browsers are used at the user front end, a cloud is used as a computing server, and all necessary controls are done at the gateway server at the middle layer. One important goal of the BioVLab infrastructure is to develop and implement a novel system design principle for genome analysis by utilizing the *virtual collaborative lab*, a suite of tools that give a team of scientists the ability to orchestrate a sequence of data analysis tasks using remote computing resources and data storage facilities “on-demand” from the scientists’ desktop computers. The virtual lab also addresses how to dynamically configure a virtual system tailored to a specific research project by combining multiple computational tools and resources using the drag-and-drop workflow composer and genome data type. The system design principle is to provide a new, effective paradigm for small biology research labs to handle the ever-increasing amount of biological data. The current BioVLab uses Amazon EC2 and S3 as cloud computing and XBaya as a graphical workflow composer.

#### The three layer architecture in BioVLab

**Figure 1:**
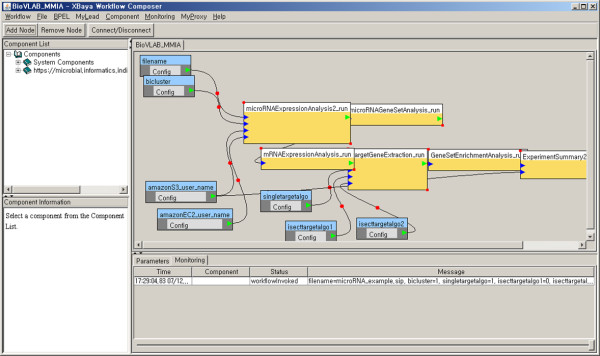
**BioVLab-MMIA workflow execution in XBaya.**

**Figure 2:**
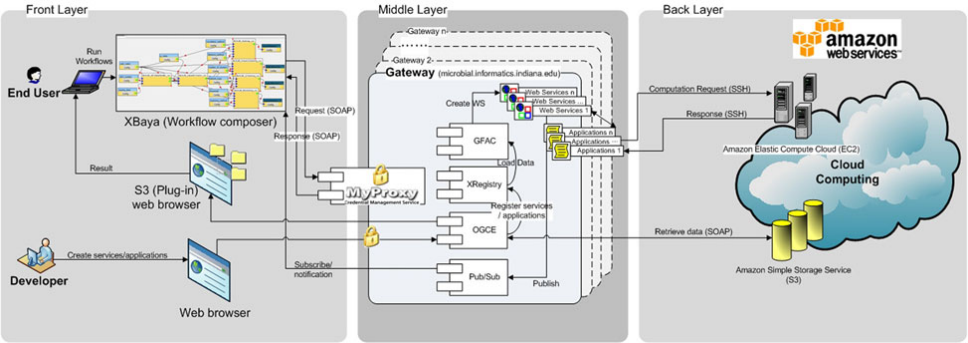
**Three-layered architecture of BioVLab system.** The first layer is XBaya, graphical workflow composer, the second layer is gateway, and the third layer is cloud environment.

The first layer, XBaya is a graphical workflow engine that enables the composition and management of scientific workflows on a desktop. The XBaya window consists of four panels as shown in Figure 1. On the top left is a panel for resources that are pre-assembled in connection with a gateway server. XBaya’s enhanced user interface simplifies composition of a workflow using scientific applications by drag-and-dropping tools and databases on the top left resource panel. Information for each resource component in the resource panel is displayed in the left bottom panel. These resource components, tools and databases are compiled for specific analysis task. Selection of these resources often requires expert knowledge for the analysis task. The compilation of these resources can help users or the workflow composer to focus only on resources that are needed for the task. By selecting a resource in the resource panel and drag-and-dropping each resource to the top right workflow panel, the user can easily compose a workflow using the resources in the resource panel. In the workflow panel, the output from a resource icon can be input to another resource icon if the input and output types are compatible. The bottom right panel is a console that monitors the execution of a workflow, showing which application (icon) is being executed and also ensuring the execution of each application by repeatedly trying the execution of each application if not successful. The execution is monitored in connection with the gateway server in an asynchronous way so that a long running workflow can be monitored. XBaya sends a request for the computation to the middle layer, and then the gateway in turn ensures the execution of an application on the cloud using the Web Service Definition Language (WDSL). The modular architecture of BioVLab is built on top of the Open Grid Computing Environments (OGCE) toolkit, that provides a web interface, database, and pub/sub system. Middle layer is a gateway that consists of Gfac, XRegistry, OGCE, and the Pub/Sub system. All resource components used in the workflow for a certain project should be registered at the gateway using XRegistry. Gfac is an application factory service for wrapping command-line tools as Web services. Utilizing the Gfac and Xregistry services, BioVlab allows users to integrate their own tools to the system or to include existing tools for the analysis task. This way, Biovlab handles system extensibility issues. The Pub/Sub system is a publish-subscribe based message broker implemented on top of Apache Axis2 web services stack. It implements the web service eventing and web service notifications specifications and incorporates a message box component that facilities communications with clients behind firewalls and overcomes network glitches (http://airavata.apache.org/). Figure 2 shows the illustration of the three-layered architecture of the BioVLab system.

#### Bio/medical cloud environment with BioVLab

On top of the BioVLab infrastructure, several systems have been built and they are available for researchers. For a specific research task, all necessary tools and databases are pre-installed as a virtual machine image. The availability of such machine image removes the user’s burden to collect and install resources and compose a workflow. In addition, the loaded biological big resources by the cloud vendors makes cloud-based systems easy to use. A workflow is pre-composed using the resources so that users do not have to survey, locate, install computational tools and databases. This way, a cloud environment for a specific science project is constructed and served to the public. Having the cloud environment for a specific science project, scientists can easily perform an analysis task by simply downloading a pre-composed workflow, creating an account on the Amazon cloud, and executing the workflow with the user’s own data. This download-workflow-and-execute computing model is a very easy to use cloud service for a specific science project. In addition, an expert user can modify the pre-composed workflow using the Xbaya workflow composer. Biovlab handles the usability issue by providing the download-and-execute pre-composed workflow model and the extensibility issue by utilizing a workflow where the user can modify the pre-composed workflow using pre-collected resources.

Two systems, BioVLab-MMIA and BioVLab-mCpG, are developed using the BioVLab infrastructure.

MMIA (MicroRNA and MRNA Integrated Analysis) [29] is a web-based analysis tool for the integrated analysis of microRNA and mRNA expression data. The complexity of the analysis comes from the fact that two different genetic elements are of different types and also from the fact that information about their relationship of microRNA’s target genes are inaccurate. Since microRNA degrades their target genes, MMIA utilizes inversely correlated expression patterns between microRNA and mRNA (perfect seed-pairing between microRNA and mRNA is associated with mRNA destabilization) and reports the results of two computational analyses. Migrating the functionality of MMIA to BioVLab as a biological cloud environment was recently done. Based on the architecture of BioVLab, MMIA’s analysis is performed over the cloud, which was formerly done locally or over a server. Expression data of microRNA and mRNA are uploaded to the cloud via the Amazon S3 Interface, which are analyzed and reported back to the user. The reference genome data, hg18, is pre-loaded on the cloud, which is downloaded from the UCSC genome browser. Once users have the input files on Amazon S3, access control for the Amazon S3 bucket (ACLs) is prepared for analysis execution. Parameter configuration, including workflows, for the analysis may be done via the web page generated by the Amazon S3 component for the specific user. The results are provided in the Amazon S3 buckets, which are accessible by clicking the View icon in the XBaya workflow or through the web browser. BioVLab-MMIA cloud is available as Amazon Machine Id: ami-957291fc.

BioVLab-mCpG [30] performs integrated analyses of DNA methylation, sequence variation, and gene expression for phenotypically distinct cell lines. Methylation profiling of genomes and sequence variation detection (i.e., SNPs) on a genome scale can be routinely done using next generation sequencing and microarray technologies. An integrated analysis of global methylation, SNPs, and gene expression profiles would have the potential to reveal biological relationships between altered cytosine methylation, sequence variations, and mRNA levels. In BioVLab-mCpG, pre-composed workflows for the integrated analysis are performed, beginning with differentially expressed genes, differentially methylated regions, and information theoretic-integrated analysis of DNA methylation and sequence variation data. Furthermore, the integrated analysis can be performed using input gene sets for specific biological pathways of interest. BioVLab-mCpG is implemented using R, Perl, and Python programming languages and utilizes Samtools [31], the MACS peak finding package [32], and the Bioconductor package [33]. By deploying the package on the Amazon EC2 Cloud Computing as an Amazon machine image, users can simply use the image to perform the integrated analysis, thereby ensuring that the integrated analysis package is readily available to the entire research community.

### Standardization and security

In addition to cloud features that we have discussed so far, there are remaining important issues for bio/medical cloud systems, such as standardization and security. Without resolving these issues, many companies and research facilities can hardly use cloud systems even if cloud systems become powerful enough to handle bio/medical big data. Standardization of tools and data formats is required for massive and parallel analysis of bio/medical bio data. Otherwise analysis tasks of multiple types can be hardly composed and performed by mundane users.

Security is the most sensitive issue since biomedical data has personal information that has legal and ethical repercussions. Because a cloud system is a third-party service provider, users may not be comfortable allowing expensive pre-analyzed and personal data stored in cloud systems. Appropriate authentication for the entire analysis process, while providing automatic execution of multi-step analysis tasks, is very important. BioVLab uses MyProxy from the TeraGrid project for authentication. However, this is still primitive. Recently there has been interesting approach that uses a novel hybrid MapReduce on the private and public cloud [34] by partitioning genome sequence data on the public and private spaces where computationally intensive tasks are performed on the public cloud using encrypted data and computation with sensitive data is performed on the private cloud utilizing computational results on the public cloud.

We believe that there will be more interesting approaches for standardization and security issues on the cloud.

## Conclusions

The analysis of big data in the bio and medical fields requires very big computing power and many research facilities and clinics are unable to afford such computing facilities. Thus cloud computing has gained great interest in the bio and medical fields to analyze big data and a growing number of bioinformatics cloud systems have been developed. In this paper we surveyed current bioinformatics cloud systems and discussed design issues. To handle big data of multiple types, analysis tasks require use of appropriate tools and databases from a vast number of tools and databases. Thus simply using cloud computing does not resolve the issue of big data analysis in the bio and medical fields. We discussed this issue by introducing a concept of cloud environment for specific scientific research goals.

## Authors’ information

Chae, H. is a Ph.D candidate in Computer Science Department, School of Informatics and Computing, Indiana University at Bloomington (IUB). After receiving his M.S. degree in Computer Science in 2007 from IUB, his research interest has lain mainly in Systems in Bioinformatics. He recently joined Bioinformatics Institute in Seoul National University (SNU) as a researcher and working in development of cloud-based biological systems.

Jung, I. received his M.S. degree from the Department of Computer Science at Yonsei University in 2007 with a major interest in sensor networks. He has been with LG Electronics as a research engineer for 4G LTE and WiMAX standards. He know has interest in bioinformatic technologies and is currently researching at the Interdisciplinary Program in Bioinformatics in Seoul National University.

Hyungro Lee received BSc degree from the Sungkyunkwan University, Seoul, Korea, in 2007 and MSc degree from the Indiana University, Bloomington, IN, in 2010. Since September of 2010, he is following a doctoral track in Computer Science at Indiana University Bloomington and he is working as a researcher of the FutureGrid project, an NSF funded significant new experimental computing grid and cloud test-bed to the research community, together with user supports. His research interests are parallel and distributed systems, and cloud computing.

Suresh Marru (http://people.apache.org/~smarru/) directs the Science Gateways program within the NSF funded Extreme Science and Engineering Discovery Environment (XSEDE). In this role he is responsible for specialized scientific community interfaces to large scale cyberinfrastructure. Marru is also the principal research systems architect within Pervasive Technology Institute of Indiana University and is a founding member of the Apache Airavata project management committee. Marru’s current research focuses on working alongside multidisciplinary experts in the field of eScience to developed open community driven web and service-based scientific workflow and gateway systems for individual research, collaboration, outreach, and cross-disciplinary collaboration. These systems assist scientists to focus on their research without the need to be distracted my emerging information technology and cyberinfrastructure.

Seong-Whan Lee is the Hyundai-Kia Motor Chair Professor at Korea University, where he is the head of the Department of Brain and Cognitive Engineering and the director of the Institute for Brain and Cognitive Engineering. He received the B.S. degree in computer science and statistics from Seoul National University, Seoul, Korea, in 1984, and the M.S. and Ph.D. degrees in computer science from Korea Advanced Institute of Science and Technology in 1986 and 1989, respectively. His research interests include pattern recognition, computer vision, and brain engineering. He has more than 250 publications in international journals and conference proceedings, and authored 10 books.

Sun Kim is an associate professor at Seoul National University and director of Bioinformatics Institute in Seoul National University (SNU). Before joining SNU, he was Chair of Faculty Division-C, Director of Center for Bioinformatics Research, an Associate Professor in School of Informatics and Computing, an Adjunct Associate Professor of Cellular and Integrative Physiology, Medical Sciences Program at Indiana University (IU) - Bloomington. Prior to IU, he worked at DuPont Central Research from 1998 to 2001, and at the University of Illinois at Urbana-Champaign from 1997 to 1998. Sun Kim received B.S and M.S and Ph.D in Computer Science from Seoul National University, Korea Advanced Institute of Science and Technology (KAIST), and the University of Iowa respectively. He is on the board of directors for ACM SIG Bioinformatics, vice chair for education at IEEE Technical Committee on Computational Life Sciences (TCCLS), co-editor for International Journal of Data Mining and Bioinformatics, and co-organizing many scientific meetings including ACM Conference on Bioinformatics, Computational Biology, and Biomedicine 2011, IEEE International Conference on Bioinformatics and Biomedicine (BIBM) 2008 and 2009.
